# *Echinococcus* spp. Tapeworms in North America

**DOI:** 10.3201/eid2402.161126

**Published:** 2018-02

**Authors:** Jacey Roche Cerda, Danielle Elise Buttke, Lora Rickard Ballweber

**Affiliations:** Colorado State University, Fort Collins, Colorado, USA (J.R. Cerda, L.R. Ballweber);; National Park Service, Fort Collins (D.E. Buttke)

**Keywords:** taeniid, helminth, tapeworm, cestode, zoonoses, canids, wolves, coyotes, deer, elk, parasites, North America, Echinococcus

## Abstract

Alveolar and cystic echinococcosis are emerging and reemerging in Europe, Africa, and Asia. The expansion of *Echinococcus* spp. tapeworms in wildlife host reservoirs appears to be driving this emergence in some areas. Recent studies suggest a similar phenomenon may be occurring in North America. We describe the context of *Echinococcus* spp. research in North America, with a specific focus on the contiguous United States. Although studies were conducted in the United States throughout the 1900s on various sylvatic and domestic *Echinococcus* spp. tapeworm cycles, data are lacking for the past ≈30 years. We review previous research, provide analysis of more recent focal studies, and suggest that *Echinococcus* spp. tapeworms, in particular *E. canadensis*, may be underrecognized. As a result, we suggest that additional research and surveillance be conducted for these tapeworms in wildlife host reservoirs across the United States.

*Echinococcus* spp. (family Taeniidae, class Cestoda) are zoonotic tapeworms currently infecting 2–3 million persons worldwide and causing US $200–$800 million in annual economic losses related to human infection (*1**,**2*). Infection appears to be increasing, reemerging, and geographically expanding in multiple locations across Europe, Asia, Africa, and the Americas (primarily in Latin America), with >200,000 new cases/year (*3*). *Echinococcus* spp. tapeworms have complex domestic and sylvatic life cycles that affect the health of >40 companion animal, livestock, and wildlife host species (*4**,**5*).

The basic *Echinococcus* spp. life cycle involves 2 hosts, where carnivores (wild and domestic) are the definitive hosts and small mammals and ungulates (domestic and wild) are the intermediate hosts (*6*). From within the small intestine of the definitive host, the mature tapeworm releases immediately infective eggs that are shed with the feces into the environment. Intermediate hosts ingest the eggs as they feed on contaminated vegetation. Once ingested, the oncosphere hatches and penetrates the small intestine to migrate to various organs and tissues, where it develops into one or more hydatid cysts (*6*). Definitive hosts ingest the cysts when feeding on the viscera of infected intermediate hosts (*6*). Humans are aberrant dead-end hosts that are infected from accidental ingestion of eggs, typically from interaction with domestic dogs, which act as bridge hosts between wildlife and the human environment. Contamination of the human environment may occur either directly (from feces) or indirectly (eggs carried on paws and fur) (*7*). Humans may also become infected through foodborne transmission, most often through eating inadequately washed fruits and vegetables (*8*). Intermediate hosts and humans may develop alveolar, cystic, or polycystic echinococcosis, depending on the parasite species involved (*6*). 

Infection in livestock can cause substantial economic losses, including the condemnation of infected viscera; decreased meat, milk, and wool production; and delayed fecundity, growth, and performance (*4*). Estimated global economic loss due to infection in production animals is US $1.5–$2 billion annually (*2*). The global disease burden on wildlife species is unknown, as is the effect that echinococcosis may have on the overall fitness of wildlife animals and populations.

Throughout the early decades of the 1900s, only 1 species of *Echinococcus*, *E. granulosus*, was formally recognized in North America and across the world. Analysis of genetic and phenotypic data, host specificity and preference, and differences in human pathogenicity and tissue tropism has since revealed that *E. granulosus* sensu lato is actually composed of a complex of 10 specific genotypes, G1–10 (*9*). Subsequently, several of the genotypes or genotype complexes have been elevated to distinct species: *E. granulosus* sensu stricto (G1–G3), *E. equinus* (G4), *E. ortleppi* (G5), *E. intermedius* (G6–G7), and *E. canadensis* (G8–G10) (*9**,**10*). Genotypes 6–7 are still often grouped with *E. canadensis*; however, distinct host preferences and genetics demonstrate the proper elevation to their own species (*10*). Additional recognized species of *Echinococcus* are *E. shiquicus*, *E. vogeli*, *E. felidis*, *E. oligarthra*, and *E. multilocularis*. *E. granulosus* sensu stricto and *E. multilocularis* tapeworms cause the 2 most pathogenic forms of disease in humans; *E. multilocularis* infections are the most deadly, given the metastatic nature of the cysts and the related difficulty of treatment.

Although well studied globally, the current presence, prevalence, and transmission dynamics of *Echinococcus* spp. tapeworms in the contiguous United States are currently unknown. Substantial research was conducted between the early 1930s and the 1980s; however, very little research has occurred in the past 3 decades. Given the documented expansion of these tapeworms across many regions of the world, including within Canada, a better understanding of the presence, prevalence, and disease ecology in the United States is needed, particularly because echinococcosis is not a reportable disease. Additional research will provide scientists, veterinary and human medical professionals, and other public health officials with a more complete picture of *Echinococcus* spp. cycles in the United States. Here we set the context for additional research through a synopsis of *Echinococcus* spp. and echinococcosis in humans in North America, with a focused discussion of endemic US *Echinococcus* spp. cycles from the early 1900s to the present. We conclude with recommendations for further research.

## *Echinococcus* spp. Tapeworms and Echinococcosis in Alaska and Canada

Even before advances in molecular genetics, researchers recognized the form of *E. granulosus* cycling between wild canids and their ungulate prey species in Alaska and Canada as distinct and referred to it as the northern biotype (*11*). The northern biotype species is now recognized as *E. canadensis*. In the boreal regions of North America, *E. canadensis* are ubiquitous parasites of wild canids and ungulates from the western coast of Alaska through all territories and provinces of Canada, exclusive of its east coast (*11**–**13*). Caribou (*Rangifer tarandus*), moose (*Alces alces*), and elk (*Cervus canadensis*) appear to be the most important intermediate hosts, although other ungulate species may become infected. Wolves (*Canis lupus*) and domestic dogs are the most important definitive hosts; however, coyotes (*Canis latrans*) are also competent (*11*). Rausch’s (*11*) comprehensive review of *E. canadensis* tapeworms provides extensive information regarding the strongly endemic cycles he and others observed from the 1950s to the late 1990s throughout Alaska, Canada, and other Arctic countries. Likewise, readers are directed to Schurer et al.’s (*14*,*15*) reviews of ungulate and wolf infections.

All 3 reviews indicate fairly stable *E. canadensis* cycles and transmission dynamics in the Arctic and sub-Arctic regions of North America. For example, Rausch reported that in a randomly collected sampling of 200 wild canids in the Brooks Range of Alaska, ≈30% were infected with *E. canadensis* tapeworms. This finding is similar to Schurer et al.’s (*15*) recent report indicating infections in 37% (71/191) of wolves sampled across Canada. Rausch (*16*) found 24% (24/101) of moose infected in an agricultural region of southern Alaska, and 4% (1/23) of moose infected in the Anchorage area. Schurer et al.’s (*14*) review of ungulate infections in Canada revealed prevalence of 0%–73% in elk, 1–21% in caribou, 11–38% in moose, and 0.3%–44% in white-tailed deer (*Odocoileus virginianus*), depending on sample location.

*E. multilocularis* tapeworms are similarly endemic in Alaska and Canada, spanning from the northern Arctic regions south into the rural and urban southern provinces of Canada, where urban coyotes and domestic dogs have been confirmed as both definitive and aberrant intermediate (dog) hosts (*7**,**13**,**17**,**18*). As with *E. canadensis*, Rausch et al. (*19*) performed important work related to *E. multilocularis* in Alaska, with a particular focus on St. Lawrence Island. Their work revealed heavy infections in Arctic fox (*Vulpes lagopus*) and their small mammal prey species. The mean rate of infection for 1,579 fox examined was 77%, whereas infection in prey animals could range from 10% to 80% (*19*). On St. Lawrence Island, dog necropsies also revealed a 12% prevalence of infection (*20*).

Gesy et al.’s (*21*) recent review of *E. multilocularis* tapeworms across Canada demonstrated coyotes, red fox (*Vulpes vulpes*), wolves, and Arctic fox were all competent hosts, with prevalence differences related to sampling location. For example, their report indicated 37% (10/27) of coyotes and 17% (1/6) of red fox were infected in Quesnel, British Columbia (*21*). Three dogs in Canada were also recently found to be infected with cysts (*7**,**13**,**17*). The high prevalence in wild hosts along with the new intermediate infections in urban pet dogs has increased concern that the geographic reach of *E. multilocularis* tapeworms is expanding within Canada. As a result, Massolo et al. (*22*) suggest that much more research is necessary to understand potential public health risks associated with alveolar echinococcosis (AE) in North America.

Both AE and cystic echinococcosis (CE) have been reported in Alaska and Canada, with most infections occurring in Native American populations in Alaska. During 1940–1990, a total of 300 cases of CE were reported in Alaskan Native Americans, with only an additional 3 cases between 1990 and 1999 (*23*). Pathogenicity of CE in Native American patients appeared to be fairly benign, with smaller, asymptomatic cysts that often resolved without the need for surgery (*23*). However, Castrodale et al. (*23*) reported 2 unusually severe cases of CE in Alaska in 1999, in a 51-year-old Caucasian woman and a 17-year-old Native Alaskan woman. The cysts from the latter patient were confirmed as belonging to the species *E. canadensis* (G8) (*23*). Most AE cases in Alaska also arose in Native American populations, with St. Lawrence Island as an infection hotspot. Jenkins et al. (*13*) reported 54 human cases during 1947–1986, none during 1986–2010, and potentially 5 during 2010–2014, although the latter infections were more likely to be CE.

Human AE and CE cases in Canada are much less prevalent than those in Alaska. Serosurveillance of indigenous populations in the Saskatchewan, Nunavut, Quebec, and the Northwest Territories of Canada indicated exposure to CE of 0%–48% (*20*). CE infections in nonindigenous patients are rare and often only incidentally reported. AE cases in Canada are even more rare, with 1 autochthonous case reported before 2013 (*20*). Subsequent reviews have found 12–16 additional cases; however, Deplazes et al. (*20*) suggest these numbers are under representative, given the strongly endemic regions present in Canada.

## *Echinococcus* spp. Tapeworms and Echinococcosis in Mexico

Far fewer studies have researched the *Echinococcus* spp. tapeworms present in Mexico; however, the primary species appears to be *E. intermedius* (G7), cycling between dogs and pigs (*20*). The species *E. granulosus* s.s. (G1) and *E. ortleppi* (G5) were also previously reported (*20*). Prevalence in slaughtered pigs is low, with reports of 0.27%–6.5%. Deplazes et al. (*20*) provide more extensive information regarding infections in Mexico.

## *Echinococcus* spp. Tapeworms and CE in the Contiguous United States

### Domestic Cycles

Historically, several genotypes of *E. granulosus* s.l. tapeworms were considered endemic and regularly cycling in the Mississippi Valley, the mid-Atlantic states, and multiple western and southwestern states. Franklin and Ward (*24*) reported that hydatid cysts were found in hogs and cattle in Virginia, Oklahoma, Arkansas, and Louisiana before 1951. They then reported the first discovery in Mississippi, finding that 2 of the 50 dogs they examined in 1951 were infected with *E. granulosus* s.l. tapeworms. Subsequently, Ward (*25*) reported that 6% of 8,066 slaughtered hogs in Mississippi were infected in 1956 and that 4 of the 9,300 dog intestines he examined over 8 years were infected with 300–700 adult *E. granulosus* cestodes each. At that time, 15 dogs had been confirmed with *E. granulosus* infection, with 5 of them from Mississippi (*25*). Given our current genetic understanding of *Echinococcus* spp., the species cycling in Mississippi was most likely *E. intermedius* (G7). Infections were also reported in dogs from New York, Kentucky, Georgia, and Tennessee, as well as Washington, DC (*25*). However, we were unable to find the originally cited papers to report the intermediate hosts involved and thus cannot comment on what species were present in those states.

Throughout the 1900s, an endemic dog/sheep cycle of CE also existed in the Central Valley of California. Through examination of the organs of 22,720 sheep carcasses during December 1967–June 1968, Sawyer et al. (*26*) found 1,100 infected sheep. Tracebacks conducted where possible demonstrated that infections could range from 5% to 99% of a producer’s entire herd (*26*). Some of the infected herds originated in Idaho and Utah, while the rest originated from 8 ranches in 4 California counties (*26*). For the producers with the highest herd prevalence, Sawyer et al. (*26*) investigated the ranches, took medical histories of the families, tested the onsite dogs for taeniids through arecoline purging, and tested live sheep serum for evidence of infection. Resident domestic dogs and sheep were infected on 3 of the 4 ranches evaluated, implying local transmission. Local transmission indicates that the dogs were probably allowed to consume viscera of infected sheep, with subsequent egg contamination of the human environment through dog infections (*27*). Across the same 4 ranches, 3 humans also harbored hydatid cysts; however, only 1 of these cases was confirmed as acquired within California.

In Utah, Loveless et al. (*28*) investigated dog and sheep infections during 1971–1976, finding an overall prevalence of 11.3% (95/839) in dogs and 9.8% (877/8,994) in sheep. A subsequent study by Anderson et al. (*29*), with data covering 1971–1982, revealed a prevalence of 9.7% (109/1,120) in dogs and 7.1% (1,116/15,775) in sheep. During 1944–1980, Utah had the highest density of autochthonous human cases in the United States, with 50 persons infected (*28*). A similar dog/sheep cycle was established in New Mexico and Arizona (*30*). In those areas, human infections were reported during 1969–1976, revealing 21 confirmed autochthonous cases. Nineteen of the cases occurred in Native Americans of the Navajo, Zuni, and Santo Domingo tribes (*30*). The source of infection in all of these areas appeared to be contamination of the human environment by infected dogs. Risk factors for human infection included sheepherding behavior; companion-animal ownership; home slaughter of sheep; allowing dogs to consume raw viscera; and contact with dogs, sheep, and swine (*31**,**32*). Although not confirmed, our current understanding of *Echinococcus* spp. tapeworms suggests that the organism causing the California, Utah, New Mexico, and Arizona cycles was most likely *E. granulosus* s.s*.*

More recent focal studies have revealed that cycles of *E. granulosus* s.l. continue to occur throughout the United States. For example, in 1994, Hoberg et al. (*33*) reported an unusual case of 3 cysts in a 14-year-old Thoroughbred horse that lived in Virginia and Maryland. The cysts were not sequenced to determine the genotype; however, those authors assert it was probably related to the species *E. equinus* (G4), as previously found in horses in Europe and Asia.

### Sylvatic Cycles

Throughout the mid-1900s to late 1900s, a sylvatic coyote/deer cycle occurred concurrently with the domestic sheep/dog cycle in California (*34**,**35*). Brunetti and Rosen (*34*) analyzed 2,049 deer carcasses collected between 1945–1969 and found an overall infection rate of 1.3%. The infected animals were concentrated in 8 counties in the Central Valley of California. Although unknown, the species involved in the sylvatic cycle may have been *E. canadensis* given the host species involved.

Riley (*36*) reported a sylvatic cycle between moose and wolves in Minnesota in the 1930s. Ramsey (*37*) reported that *E. canadensis* infection was found in a moose in northwestern Montana during 1976–1983. In 2009, Foreyt et al. (*38*) reported the presence of *E. canadensis* infections in wolves, elk, mule deer (*Odocoileus hemionus*), and a mountain goat (*Oreamnos americanus*) in Idaho and Montana; however, the specific genotypes (G8 or G10) were not delineated. In 2012, Lichtenwalner et al. (*39*) described the finding of an *E. canadensis* (G8)–infected moose in Maine.

The several sylvatic cycles reported in California, Montana, Idaho, and Minnesota indicate that *E. canadensis* tapeworms have been sporadically endemic in wild canid/ungulate populations across the United States since the first discovery in the early 1900s. With no further cases reported, the coyote/deer cycle appears to have ended in California. However, the cestode now appears to be reemerging in a wolf/ungulate cycle in the western United States.

## *E. multilocularis* and AE in the Contiguous United States

*E. multilocularis* tapeworms are also historically endemic in several northern states. During 1965–1969, Leiby et al. (*40*) examined 7,898 definitive and intermediate host mammals from eastern Montana, North and South Dakota, Minnesota, and Iowa. Among the definitive hosts, the authors found that 8.5% (131/1,540) of red fox and 4.1% (7/171) of coyotes examined were infected. In North Dakota, 55.3% of red fox were found infected in 1965, while only 7.3% were infected in 1968. No domestic cats (n = 35) or dogs (n = 88) were found to be infected (*40*). However, in a subsequent report, Leiby and Kritsky (*41*) reported that 2 adult house cats from a homestead in North Dakota were infected with *E. multilocularis* tapeworms in 1971.

Of the numerous potential intermediate hosts examined by Leiby et al. (*40*), only 3 species, the deer mouse (*Peromyscus maniculatus*), meadow vole (*Microtus pennsylvanicus*), and house mouse (*Mus musculus*), were found to have *E. multilocularis* cysts. Infected deer mice were found in all 5 states, with the highest number, 202/4,209 (4.8%), in North Dakota. Infected meadow voles were found only in Iowa and North Dakota, with 1.9% infected (20/1,033). Only 1/91 (1.1%) house mice examined, also from North Dakota, was infected. The reported infections may be an overly conservative estimate because data were obtained through visual examination of the carcasses for cysts; early or latent cysts may be too small for gross visualization.

In the 1980s, Ballard et al. (*42**,**43*) extended the known range of *E. multilocularis* tapeworms into Nebraska, Illinois, and Wisconsin, further indicating the endemic cycle in the north-central United States. Ten (27%) of 36 foxes were infected in Nebraska, 4 (10%) of 40 in Illinois, and 6 (8.3%) of 72 in Wisconsin (*42*). In the mid-1990s, the known range expanded again when *E. multilocularis* infection was found in Michigan and Ohio (*44**,**45*).

In 2000, Hildreth et al. (*46*) reported the results of a combined study of both wild canids and human trappers in South Dakota. The intestines of foxes trapped between 1987–1991, and of coyotes trapped between 1990–1991, were examined for the presence of *E. multilocularis* tapeworms*.* Results showed that 74.5% of foxes and 44.4% of coyotes were infected at the time of trapping. Because trappers are a population at extreme risk for AE, given their constant and extended contact with potentially infected wild canids, blood samples were obtained from 115 attendees of the South Dakota trappers meetings in 1990 and 1991. However, despite their high risk, none of the trappers showed evidence of infection.

Only 1 autochthonous case of AE has been confirmed in the contiguous United States, and that occurred in a 56-year-old woman from Minnesota (*47*). The patient had never lived outside of Minnesota, and her travel history included only California, Hawaii, Florida, and Manitoba, Canada. Gamble et al. (*47*) asserted that she was most likely to have been infected through interaction with pet dogs allowed to consume rodents on the farm where she grew up.

## Conclusions and Recommendations

In the past 3 decades, there has been little focus on *Echinococcus* spp. tapeworms in the contiguous United States in humans or animals. The apparent lack of concern regarding the potential public health threat posed by echinococcosis is probably due to the rarity of reported human cases. Similarly, since the 1980s, there have only been a handful of reports on the presence and prevalence of *Echinococcus* spp. tapeworms in domestic and wild animals in the lower 48 states. The lack of human cases may reflect low exposure to infected definitive hosts of either domestic or sylvatic origin. Alternatively, it may reflect lack of detection, misdiagnosis, or failure to publish literature regarding new AE and CE cases. Regardless of the reason, however, there is a legitimate concern that the public health risk may increase within the United States in the future, given the continued expansion of human infection in Europe and Asia, as well as an apparent expansion of the range of *E. multilocularis* tapeworms in Canada.

Current surveillance and basic scientific understanding of *Echinococcus* spp. tapeworms and echinococcosis in the United States is particularly lacking. In addition, there are many outstanding questions, including the presence, prevalence, and geographic distribution of *Echinococcus* spp. tapeworms across the contiguous United States; geospatial, ecologic (both biotic and abiotic), and host population variables influencing sylvatic echinococcosis dynamics; and perhaps most important, what risk these sylvatic and domestic cycles pose to human health. As noted by Massolo et al. (*22*), Canada appears to be experiencing an increase in the presence and prevalence of *E. multilocularis* tapeworms, with spillover events starting to occur. If this asserted expansion is true, a similar expansion may be occurring in the United States. We support increased surveillance of *Echinococcus* spp. tapeworms in the United States to answer these questions and in particular to focus on the existing and potential public health risks associated with endemic *Echinococcus* spp. tapeworms. To enhance data collection on current and past cases, and further define the human burden of echinococcosis, we support and recommend the use of historically underused data sources, such as the National Inpatient Sample (*48*). We believe addressing the described data gaps is very important not only because of potential spillover from sylvatic cycles but also because echinococcosis is not reportable in either animals or humans in the United States and the United States does not screen for these types of infections in imported animals. If appropriate surveillance occurs that comprehensively analyzes current endemic cycles, then effective and efficient detection of new or expanding cycles or spillover events would be much more likely to occur, leading to a decrease in potential human cases or other negative public health outcomes.
